# Has Embase replaced MEDLINE since coverage expansion?

**DOI:** 10.5195/jmla.2018.281

**Published:** 2018-04-01

**Authors:** Michael Thomas Lam, Christina De Longhi, Joseph Turnbull, Helen Rose Lam, Reena Besa

**Affiliations:** Undergraduate Student, Bachelor of Medical Science Program, Western University, London, ON, Canada; Information Specialist, Library Services, Sunnybrook Health Sciences Centre, Toronto, ON, Canada; Assistant Professor of Mathematics, Department of Economics, Business, and Mathematics, King’s University College at Western University, London, ON, Canada; Undergraduate Student, Bachelor of Social Work Program, Faculty of Liberal Arts and Professional Studies, York University, Toronto, ON, Canada; Information Specialist, Library Services, Sunnybrook Health Sciences Centre, Toronto, ON, Canada

## Abstract

**Objectives:**

The research tested the authors’ hypothesis that more researchers from the academic medicine community in the United States and Canada with institutional access to Embase had started using Embase to replace MEDLINE since Embase was expanded in 2010 to cover all MEDLINE records.

**Methods:**

We contacted libraries of 140 and 17 medical schools in the United States and Canada, respectively, to confirm their subscriptions to Embase 5 years before and 5 years after 2010. We searched the names of institutions with confirmed Embase access in Ovid MEDLINE and Embase to retrieve works authored by affiliates of those institutions. We then examined 100 randomly selected records from each of the 5 years before and 5 years after the Embase coverage expansion in 2010. We hypothesized that studies that used Embase but not MEDLINE would increase due to the Embase coverage expansion.

**Results:**

The number of studies that used Embase but not MEDLINE did not change between the pre-2010 and post-2010 periods.

**Conclusion:**

Our hypothesis was refuted. Studies that used Embase but not MEDLINE did not increase post-2010. Our results suggest the academic medicine community in the United States and Canada that had access did not use Embase to replace MEDLINE, despite the Embase coverage expansion.

## INTRODUCTION

Many types of biomedical research require searching the medical literature as part of the research process to understand the current evidence or knowledgebase. This is true when conducting primary research such as basic science or laboratory research, clinical research, and epidemiological research as well as secondary research such as narrative reviews, systematic reviews, and meta-analyses [1]. This is usually done using one or more purpose-built biomedical bibliographic database such as MEDLINE or Embase. Despite the advent of computerized searching offered since the early 1970s by MEDLINE (MEDLARS Online) and Embase (Excerpta Medica) [2], the top two most preferred biomedical databases for decades [3], research remains challenging due to the sheer volume of the medical literature [4, 5] and complexity of medical topics and terminologies, as well as possible errors in the databases [6].

It is widely recognized that a search in MEDLINE (free via PubMed, paid subscription via Ovid, and other interfaces) alone does not provide comprehensive coverage of the existing literature [7–9]. The Cochrane Collaboration recommends that authors of systematic reviews search at least Embase and Cochrane Central in addition to MEDLINE [10]. According to a study done in 2016, MEDLINE was the most popular database among studies that searched 1 or multiple databases [3]. MEDLINE was estimated to have contained 24.3 million records in 2015 [11].

In research studies that used multiple databases, Embase was the second most popular database after MEDLINE [3]. Embase, estimated to contain 29.9 million records near the end of 2014 [12], was also widely used for research on drug-related topics, as studies have shown the database offers better coverage than MEDLINE on pharmaceutics-related literature [13–15]. Some authors also recommended Embase when researching certain subtopics in health care, such as complementary and alternative medicine, prognostic studies, telemedicine, psychiatry, or health technology [16–20], making it a costly but versatile supplement and competitor to MEDLINE [2, 21].

Historically, MEDLINE and Embase were reported to have a coverage overlap ranging from 34% [22] to 70% [23]. In 2011, the Cochrane Collaboration claimed in version 5.1 of their handbook that MEDLINE and Embase each had approximately 1,800 unique journal titles that were not covered by the other database [10]. In 2017, Elsevier, the parent company of Embase, suggested that Embase had 2,800 unique journal titles, while MEDLINE had 2,500, with the 2 databases sharing 3,000 common titles [24]. Despite the variations among reports regarding common coverage at different times, an equivalent search in both MEDLINE and Embase has always been recognized as necessary when comprehensive coverage is required [8, 22, 25, 26].

**Figure 1:**
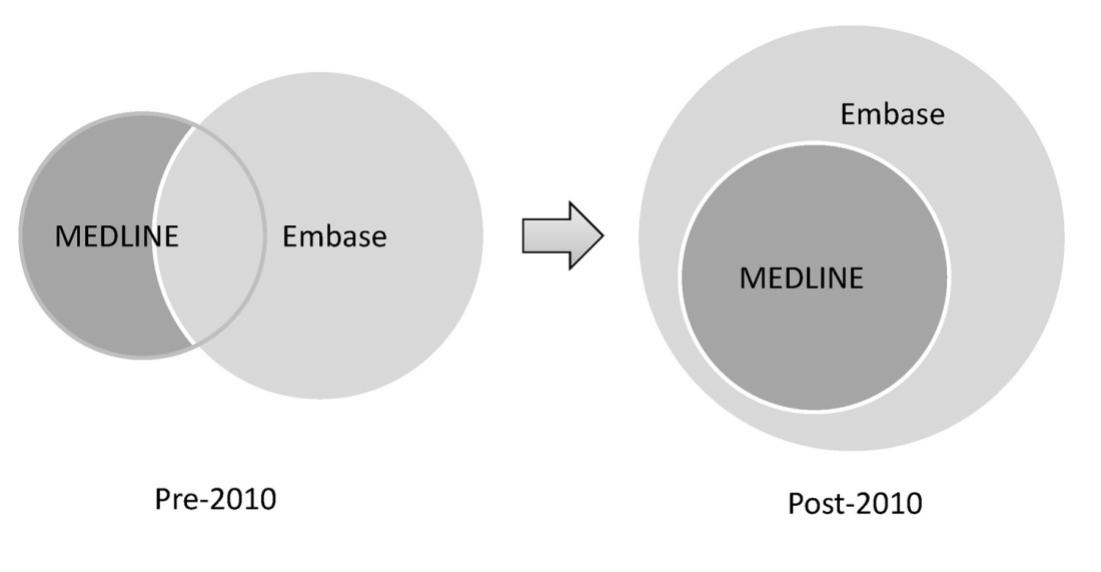
Coverage overlap of MEDLINE and Embase pre- and post-2010 ^*^ Drawings not to scale.

In 2010, Embase underwent an ambitious project to include all MEDLINE citations in Embase (Figure 1). Unique MEDLINE records are imported into Embase on an ongoing basis after mapping of Medical Subject Headings (MeSH) terms into Emtree terms. These MEDLINE records reside in Embase but remain different than regular Embase records due to the lack of original subject indexing by Embase [24]. Since this coverage expansion—at least in theory and without taking into consideration the different indexing practices of the two databases—a search in Embase alone should cover every record in both Embase and MEDLINE, making Embase a possible “one-stop” search engine for medical research. This raises the question of whether a separate search in MEDLINE is still necessary post-2010, if Embase is available to researchers via an institution-wide subscription, because one of the major obstacles to an Embase search has been its high cost [13].

The authors designed this retrospective study to test our hypothesis that a greater percentage of researchers from the academic medicine community in the United States and Canada who have access to Embase through institutional subscriptions started using Embase more frequently to replace MEDLINE since the Embase coverage expansion in 2010.

## METHODS

Between October 2016 and February 2017, we used email or telephone calls to contact the medical libraries of 117 allopathic (medical degree [MD]–granting) and 23 osteopathic (doctor of osteopathic medicine [DO]–granting) medical schools and/or their parent institutions in the United States, as well as those of 17 English- and French-speaking (MD- or doctor of medicine and master of surgery [MDCM]–granting) medical schools in Canada. There were another 19 allopathic and 15 osteopathic schools in the United States that we did not contact, as they did not yet exist in the year 2005, the year our study period began. Our purpose was to create a list of institutions that offered uninterrupted Embase access to their library users within the 5-year periods before (2005–2009) and after (2011–2015) the Embase coverage expansion in 2010.

We chose to examine studies authored by members of the academic medicine community, namely, medical schools’ affiliates, because we hypothesized that these researchers, as a group, paid the most attention to the latest evidence in the literature and received relatively strong support from professional librarians or information specialists and, hence, were more likely to make informed decisions about their choice of databases.

We then performed a search in Ovid MEDLINE and Embase in the same search session for the names of the medical schools and/or parent institutions with confirmed Embase access, directing the keyword search to the “institution (IN)” fields of only the two databases (Table 1). The strategy continued with another search for keywords such as “MEDLINE,” “PubMed,” “Embase,” “Excerpta Medica,” “literature search,” “database search,” and “literature review” in the “abstract (AB)” fields to identify studies with a literature search component. We imposed no restrictions on the publication type and included case reports, clinical trials, cohort studies, practice guidelines, narrative reviews, systematic reviews, qualitative or quantitative studies, and prospective or retrospective studies, with the aim of obtaining an overview of all research activities that used a biomedical database.

**Table 1:** Databases search strategy

	Ovid MEDLINE <1946 to April Week 1 2017>, Embase Classic+Embase <1947 to 2017 April 18>
1	(McMaster University or MacMaster University or Michael G DeGroote School of Medicine or Queens University or Queens School of Medicine or University of Calgary or Cumming School of Medicine or UCalgary or University of Alberta or UAlberta or Western University or University of Western Ontario or UWO or Schulich School of Medicine or McGill University or Universit^*^ McGill or McGill or University of British Columbia or UBC or University of Ottawa or UOttawa or Universit^*^ d Ottawa or University of Saskatchewan or USaskatchewan or Laval or Universit^*^ Laval or Laval University or University of Toronto).in. (919,463)
2	(Duke University or Duke Medicine or Emory University or Emory College or “Atlanta College of Physicians and Surgeons” or Atlanta Medical College or Atlanta School of Medicine or Southern Medical College or Georgetown University or Harvard Medical School or Harvard University or Johns Hopkins or Johns Hopkin or John Hopkins or John Hopkin or Lake Erie College or Loma Linda University or Mayo Medical or Mayo School or Mayo Clinic or Mayo Graduate School or Medical College of Wisconsin or Marquette University School of Medicine or Milwaukee Medical College or “Wisconsin College of Physicians and Surgeons” or Morehouse School or Morehouse College or New York University or NYU or Nova Southeastern University or Nova Southeastern College or Southeastern University or Southeastern College or Nova College or Nova University or NSU College or Ohio State University or Ohio State College or OSU College or University of Pennsylvania or Perelman School of Medicine or Penn Med or Penn Medicine or UPenn or Texas Tech University or Foster School of Medicine or Paul L Foster School of Medicine or Texas Tech University Health Sciences Center or Texas Tech University Health Sciences Center School of Medicine or University of Illinois or University of Illinois College of Medicine or University of Illinois at Chicago or University of Illinois Medical Center or College of Medicine at Chicago or Weill Cornell Medical College or Cornell University or Weill Cornell Graduate School of Medical Sciences or Weill Cornell Medicine or Cornell or University of Missouri Kansas City School of Medicine or University of Missouri Kansas City School of Medicine or University of Missouri Kansas City or University of Tennessee Health Science Center College of Medicine or University of Tennessee or University of Tennessee Health Science Center or Vanderbilt University School of Medicine or Vanderbilt University or Vanderbilt or Vanderbilt University Medical Center or University of Pittsburgh School of Medicine or Pitt Med or University of Pittsburgh or Rutgers or Rutgers University or Rutgers New Jersey Medical School or Rutgers Robert Wood Johnson Medical School or Robert Wood Johnson Medical School or University of Nebraska College of Medicine or University of Nebraska or Michael F Sorrell Center for Health Science Education or Sorrell Center or University of Mississippi School of Medicine or University of Mississippi or University of Mississippi Medical Center or Michigan Medicine or University of Michigan Medicine or University of Michigan or U Michigan or University of Michigan Medical School or University of Michigan School of Medicine).in. (2,564,484)
3	1 or 2 (3,439,905)
4	(MEDLINE or Pubmed or Embase or Excerpta Medica or ((literature or database or systematic^*^) adj2 (search^*^ or review^*^))).ab. (597,957)
5	3 and 4 (74,070)
6	limit 5 to english language (73,810)
7	limit 6 to yr=“2015” (10,170)
8	remove duplicates from 7 (5,606)
9	limit 8 to full text (1,133)

The search was then limited to one year at a time for the five years from 2005 to 2009 (before the coverage expansion of Embase in 2010) and the five years from 2011 to 2015 (after the expansion). We excluded the year 2010 because it was a transition period, during which the uploading of MEDLINE records into Embase was still in progress. To facilitate examination of full text, we limited our search to articles with full text available from our local university library, the University of Toronto Libraries. The resulting records from the Ovid search, for each year, were rearranged into random order using randomization software [27].

**Figure 2:**
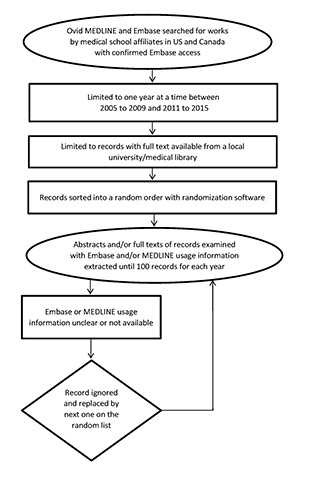
Research process flowchart

We then imported the records into EndNote X8 software. Our team carefully screened the individual abstracts of these randomized records to extract information pertaining to the usage of Embase and/or MEDLINE. If the information of interest was not found in the abstracts, we examined the full text. Records that did not clearly mention using either MEDLINE or Embase in the full text were voided and excluded until 100 informative records were covered for each year. Information about the usage of databases other than MEDLINE or Embase was ignored, as the focus was on whether researchers had used Embase to replace MEDLINE (Figure 2).

We measured three parameters for comparison between the pre- and post-2010 periods. We argue that any increase in the percentage of studies that used Embase but not MEDLINE (E) from pre-2010 (E1) to post-2010 (E2) supported our hypothesis. For reference, we also looked at the percentage of studies that used both MEDLINE and Embase (ME) and the percentage of studies that used MEDLINE but not Embase (M). While any change in these two values is of reference value, we argue that any change in these two values alone does not support or refute our hypothesis. We reason any abandonment of MEDLINE in favor of Embase would be clearly reflected as an increase in Embase but not MEDLINE (E).

## RESULTS

We compiled a list of 39 medical schools and institutions that provided uninterrupted Embase access to their library users within the period from 2005 to 2015, representing 20.5% of US MD schools (24/117), 13.0% of US DO schools (3/23), and 70.5% of Canadian schools (12/17) that existed in 2005. Medical schools with no or intermittent access to Embase during the period were excluded.

Upon careful examination of the 500 randomly selected records from each of the pre- and post-2010 periods, the value of E (Embase but not MEDLINE) decreased from 0.2% (E1=1/500) during the pre-2010 period to 0 (E2=0/500) during the post-2010 period. For reference, the value of ME (both MEDLINE and Embase) increased from 38.8% (ME1=194/500) to 51.4% (ME2=257/500), while the value of M (MEDLINE but not Embase) decreased from 61.0% (M1=305/500) to 48.6% (M2=243/500) (Table 2). We found a significant change in the distributions of the pre- and post-2010 measures of E, M, and ME over time (3 pairs of possible states compared over 2 different intervals of time, χ^2^ (2, n=500)=34.06, *p*<0.0001). Statistical sampling errors were estimated at 2% for these measures at the 95% confidence level. Thus, contrary to our initial hypothesis, we found that, within statistical error, while the value of E did not increase from pre- to post-2010, a greater proportion of researchers now use both Embase and MEDLINE, and fewer rely on MEDLINE alone.

**Table 2:** Embase and MEDLINE usage pre- and post-2010 from a random sample of records

Year	2005	2006	2007	2008	2009	Total pre-2010	2011	2012	2013	2014	2015	Total post-2010
Records retrieved	1,231	1,457	1,571	1,789	2,275	8,323	3,017	3,372	3,925	4,648	5,172	20,134
Random sample size	100	100	100	100	100	500	100	100	100	100	100	500
Searched Embase w/o MEDLINE (E)	0	1	0	0	0	1 (E_1_=1/500)	0	0	0	0	0	0 (E_2_=0/500)
Searched MEDLINE w/o Embase (M)	58	69	59	60	59	305 (M_1_=305/500)	50	50	54	41	48	243 (M_2_=243/500)
Searched both MEDLINE and Embase (ME)	42	30	41	40	41	194 (ME_1_=194/500)	50	50	46	59	52	257 (ME_2_=257/500)

To confirm the adequacy of our sample size, we completed an analysis of another 250 independent and randomly selected records from each of the pre- and post-2010 periods (50 records from each year). In this smaller sample, the value of E was unchanged at 0 during both time periods. Here, statistical sampling errors were estimated as 3% for all measures at the 95% confidence level. The shifts in the ME and M proportions were also comparable to those found for the larger sample within statistical error.

## DISCUSSION

Our results suggest that, despite the Embase coverage expansion to include all of MEDLINE since 2010, researchers in the academic medicine community of the United States and Canada with access to Embase did not use Embase to replace MEDLINE.

This could be due to several reasons. While one possibility was that researchers did not know about this major coverage change in Embase, we argue that this was improbable, as researchers in this population were likely supported by professional librarians or information specialists at their institutions. Another argument against this scenario was that there has been no gradual increase in searching Embase but not MEDLINE (E) between the years 2011 and 2015. If lack of awareness about Embase coverage expansion was the reason, as information was shared and knowledge of researchers improved with time, there should have been a gradual change, which was not observed.

Another possible explanation is that researchers and their professional librarians or information specialists favored the indexing practice and quality of MEDLINE over those of Embase, either out of preference or habit. There have been reports of EMTREE terms (subject headings used in Embase) being assigned too loosely in Embase, resulting in subject searches with unnecessarily high sensitivity and low precision [28]. It is also possible that some researchers would like to cover the MEDLINE-in-Process materials and some additional publications in PubMed that are not covered promptly or completely by Embase, even after coverage expansion [29, 30]. Another possibility is that the academic medicine community in the United States and Canada prefers a not-for-profit North American database (MEDLINE) created by a government agency over a privately owned commercial European database (Embase). The true reasons behind researchers not using Embase to its full potential are unknown and deserve further investigation.

Another trend shown in the results was a drop in M (used MEDLINE but not Embase) and an increase in ME (used both MEDLINE and Embase). Historically, most studies that searched only one database chose MEDLINE/PubMed, but this type of single database study has been in decline [3].

The results of our study suggest that researchers still view Embase and MEDLINE as separate resources to be searched individually, despite the Embase coverage expansion. Indeed, the Cochrane Collaboration did not call for a change to its recommendation for authors after the Embase expansion in 2010 [10].

We were surprised that a mere 20.5% of US MD schools and 13.0% of US DO schools provided uninterrupted access to Embase during the period from 2005 to 2015. By comparison, 70.5% of Canadian medical schools offered access during the same period. This occurred at a time when the average number of databases searched in systematic reviews was reported to be increasing, with Embase being the second-most-used resource alongside MEDLINE/PubMed [3]. The high cost of Embase and the lack of awareness of the importance of Embase among the schools’ decision-makers might be factors leading to the low subscription rate among medical school libraries in the United States.

A limitation of our study is that we restricted the search results to records with full text available from the University of Toronto Libraries system. We did this to have the capacity to examine the full text of each record, when needed, without paying for interlibrary loan orders. This might have resulted in selection bias toward studies published in relatively well-established journals that were more likely to be available in the major university medical library we accessed. Hence, our study results are representative of this particular collection and might or might not be representative of all records in the databases. Despite this limitation, however, we believe our research results are of reference value to information professionals and medical researchers.
